# Dynamic three-dimensional liver volume assessment of liver regeneration in hilar cholangiocarcinoma patients undergoing hemi-hepatectomy

**DOI:** 10.3389/fonc.2024.1375648

**Published:** 2024-04-19

**Authors:** Haoyu Zhao, Baifeng Li, Xiaohang Li, Xiangning Lv, Tingwei Guo, Zongbo Dai, Chengshuo Zhang, Jialin Zhang

**Affiliations:** ^1^ Department of Hepatobiliary Surgery, The First Hospital of China Medical University, Shenyang, China; ^2^ Department of Radiology, The First Hospital of China Medical University, Shenyang, China

**Keywords:** hilar cholangiocarcinoma, liver regeneration, bilirubin, preoperative biliary drainage, liver insufficiency

## Abstract

**Background:**

For patients with hilar cholangiocarcinoma (HC) undergoing hemi-hepatectomy, there are controversies regarding the requirement of, indications for, and timing of preoperative biliary drainage (PBD). Dynamic three-dimensional volume reconstruction could effectively evaluate the regeneration of liver after surgery, which may provide assistance for exploring indications for PBD and optimal preoperative bilirubin value. The purpose of this study was to explore the indications for PBD and the optimal preoperative bilirubin value to improve prognosis for HC patients undergoing hemi-hepatectomy.

**Methods:**

We retrospectively analyzed the data of HC patients who underwent hemi-hepatectomy in the First Affiliated Hospital of China Medical University from 2012 to 2023. The liver regeneration rate was calculated using three-dimensional volume reconstruction. We analyzed the factors affecting the liver regeneration rate and occurrence of postoperative liver insufficiency.

**Results:**

This study involved 83 patients with HC, which were divided into PBD group (n=36) and non-PBD group (n=47). The preoperative bilirubin level may be an independent risk factor affecting the liver regeneration rate (*P*=0.014) and postoperative liver insufficiency (*P*=0.016, odds ratio=1.016, β=0.016, 95% CI=1.003–1.029). For patients whose initial bilirubin level was >200 μmol/L (n=45), PBD resulted in better liver regeneration in the early stage (*P*=0.006) and reduced the incidence of postoperative liver insufficiency [*P*=0.012, odds ratio=0.144, 95% confidence interval (CI)=0.031–0.657]. The cut-off value of bilirubin was 103.15 μmol/L based on the liver regeneration rate. Patients with a preoperative bilirubin level of ≤103.15 μmol/L shown a better liver regeneration (*P*<0.01) and lower incidence of postoperative hepatic insufficiency (*P*=0.011, odds ratio=0.067, 95% CI=0.008–0.537).

**Conclusion:**

For HC patients undergoing hemi-hepatectomy whose initial bilirubin level is >200 μmol/L, PBD may result in better liver regeneration and reduce the incidence of postoperative liver insufficiency. Preoperative bilirubin levels ≤103.15 μmol/L maybe recommended for leading to a better liver regeneration and lower incidence of postoperative hepatic insufficiency.

## 1 Introduction

Hilar cholangiocarcinoma (HC) refers to cholangiocarcinoma involving the common hepatic duct, right and left hepatic ducts, and confluence. HC is also known as proximal cholangiocarcinoma or Klatskin tumor, and it accounts for 50% to 70% of all biliary tract tumors (1, 2). Patients with HC usually have a poor prognosis, with a 5-year survival rate of approximately 40% and a recurrence rate of up to 75% after resection (3, 4).

For patients with HC who are suitable candidates for surgery, the ideal treatment is resection of the extrahepatic and intrahepatic bile ducts and the involved ipsilateral liver (1). Patients with HC usually develop related postoperative complications such as liver dysfunction or liver failure, and such complications are accompanied by high mortality rates of about 10% in Western referral centers (5–7). Postoperative liver regeneration is an important repair mechanism that is attracting increasingly more attention because of its ability to lessen liver damage and avoid liver failure after hepatectomy (8, 9). The change in liver volume is a reliable way to evaluate liver regeneration. Many studies have explored the change in liver volume as an important reference index in clinical analysis (10, 11).

Patients with HC who develop severe jaundice usually need biliary drainage before hemi-hepatectomy. The high risk of complications and mortality after surgery in patients with HC are closely related to preoperative jaundice (12, 13). PBD can palliate jaundice and reduce the incidence of postoperative complications and mortality by promoting liver regeneration (14). To date, studies on liver regeneration after hepatectomy for HC have mainly been based on animal experiments; few clinical studies have been performed (15, 16). Research has shown that the relative liver weight, expression of proliferating cell nuclear antigen, DNA synthesis rate, and mitotic index are important indicators of liver regeneration (16–18). Whether patients with HC need biliary drainage and the degree and timing of such biliary drainage remain controversial (19–21). This study was performed to evaluate the postoperative liver regeneration of patients with HC and analyze the related factors affecting postoperative liver regeneration and liver insufficiency. The overall goal is to provide a reference for preoperative clinical decision-making for patients with HC.

## 2 Materials and methods

### 2.1 Patient selection

This study retrospectively evaluated the data of patients who were diagnosed with HC and underwent liver resection in the First Affiliated Hospital of China Medical University from 1 January 2012 to 1 April 2023. The records of 1317 patients were retrieved. Of these patients, we excluded 611 who only received jaundice reduction treatment or extrahepatic bile duct resection without hemi-hepatectomy, 499 with incomplete imaging or clinical data, and 124 with a computed tomography (CT) follow-up duration of >24 weeks or <1 week. This study was approved by the Institutional Review Committee of the First Affiliated Hospital of China Medical University This study was approved by the Institutional Review Committee of the First Affiliated Hospital of China Medical University (Approved number: 2024-4).

### 2.2 Definitions

HC was defined as cholangiocarcinoma arising from the common hepatic duct, left and right hepatic ducts, or confluence of the hepatic ducts, and intrahepatic cholangiocarcinoma invading the hepatic hilus (22). The Bismuth–Corlette classification was used to classify the tumors (23). Liver insufficiency was defined as a postoperative total bilirubin level of >119.7 μmol/L or, in patients with preoperative jaundice, as a total bilirubin level on postoperative day 5 to 10 that was higher than the preoperative level (5, 24, 25). In this study, the initial bilirubin was measured on the day before or most recently to PBD treatment, and the preoperative bilirubin was the bilirubin value measured on the day before or most recently to the surgery in the PBD group. Meanwhile, in the non-PBD group, the initial bilirubin value, which is equal to preoperative bilirubin value, was measured on the day before or most recently to the surgical treatment. Using the standard liver volume (SLV) formula for Chinese adults, the SLV (mL) was calculated as 11.5 × body weight (kg) + 334 (26).

### 2.3 Imaging

Three CT scanners were used in this study: Somatom Definition Flash CT (Siemens Healthineers, Erlangen, Germany), Brilliance CT (Royal Philips Electronics, Amsterdam, Netherlands), and Aquilion ONE CT (Toshiba Corporation, Tokyo, Japan). All patients underwent a plain or enhanced scan in the conventional supine position. The arterial phase, portal venous phase, and delayed phase were performed at 25 to 30s, 60 to 70s, and 160 to 180s, respectively, after the injection of a non-ionic contrast agent.

### 2.4 Segmentation

The sampled sequence of CT images was imported into a structural software application (3D Slicer, version 5.3.0; http://www.slicer.org). The volumes of interest [i.e., baseline liver volume (V_BLV_) and postoperative liver volume (V_PRO_)] were manually delineated by physicians with many years of experience in radiology. The surgical segmentations were carefully reviewed and delineated by senior physicians with 30 years of experience in radiology and hepatobiliary surgery according to the surgical records. In hemi-hepatectomy, the segmentation was performed along the middle hepatic vein; in hemi-hepatectomy combined with caudate lobectomy, the segmentation included the caudate lobe of the liver. The volume of future liver remnant after resection was defined as V_FLR_ (Future liver remnant, FLR). The rate of liver regeneration is calculated by dividing the changes between the V_PRO_ and V_FLR_ by weeks (10). These reviews were performed without access to the patients’ clinical information.

### 2.5 Statistical analysis

Numerical variables are expressed as mean ± standard deviation. Student’s t-test or the Mann–Whitney U test was used to analyze continuous variables. Categorical variables were analyzed using Pearson’s chi-square test or Fisher’s exact test. Factors associated with the liver regeneration rate were analyzed by univariate analysis and multiple linear regression analysis. The cut-off value of preoperative bilirubin was estimated by Youden’s index. Univariate and multifactorial logistic regression analyses were performed to explore the factors affecting postoperative liver insufficiency. A *P* value of <0.05 was considered statistically significant. SPSS version 27.0 (IBM Corp., Armonk, NY, USA) was used for the statistical analysis.

## 3 Results

### 3.1 Patient characteristics

**Figure 1:**
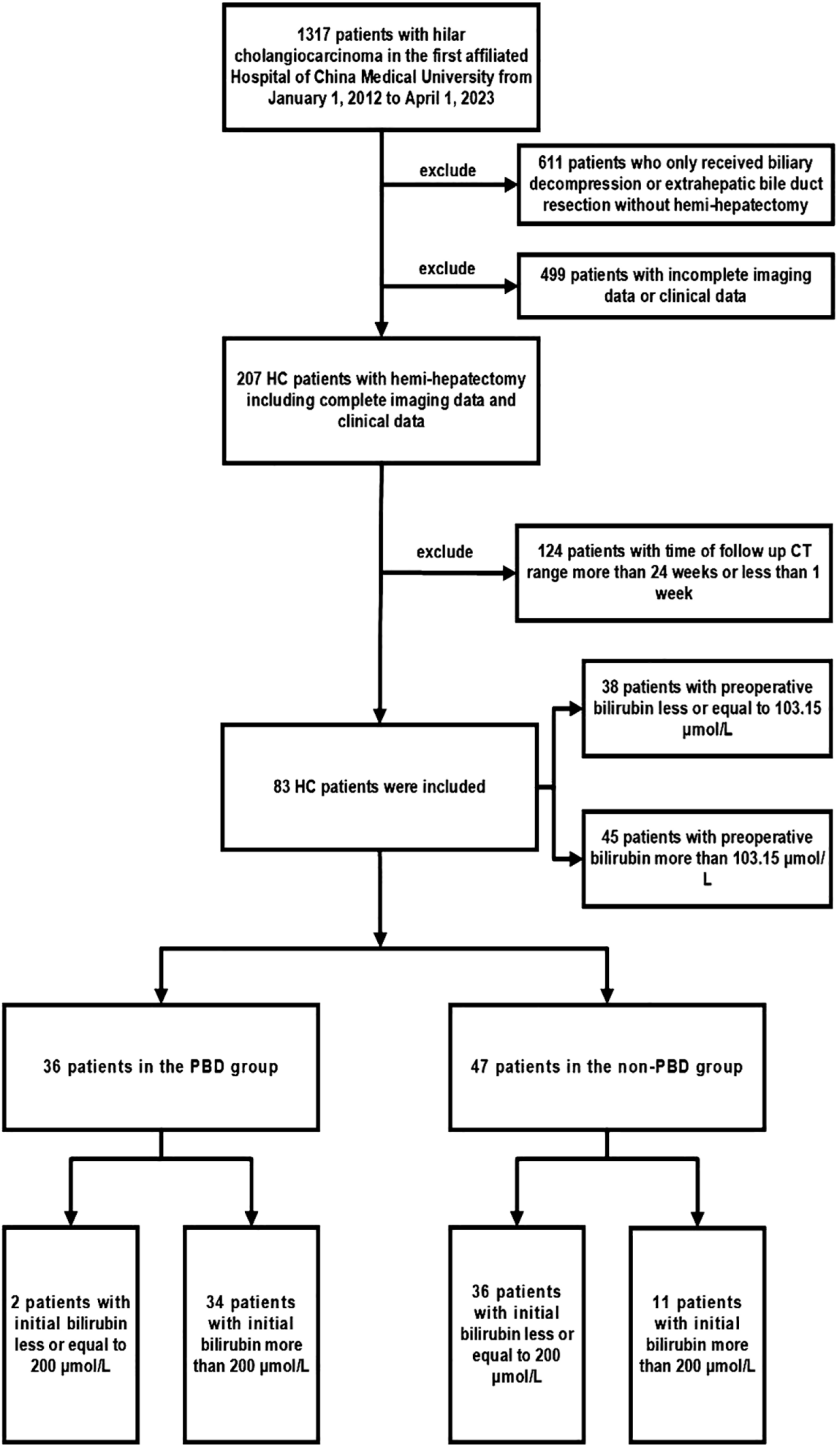
Flow chart of patient enrollment in this study.

This study involved 83 patients with HC who underwent hemi-hepatectomy. Of these 83 patients, 36 underwent hemi-hepatectomy after PBD (PBD group) and 47 underwent hemi-hepatectomy alone (non-PBD group). The patients’ mean age was 64.54 ± 9.56 years, and the male:female ratio was 52:31. The ratio of patients with an initial bilirubin level of >200: ≤200 μmol/L was 34:2 in the PBD group and 11:36 in the non-PBD group. Among all 83 patients, the ratio of patients with a preoperative bilirubin level of >103.15: ≤103.15 μmol/L was 45:38. All patients underwent hemi-hepatectomy, and the ratio of left hemi-hepatectomy or extended left hemi-hepatectomy to right hemi-hepatectomy or extended right hemi-hepatectomy was 55:28. No patients underwent preoperative portal vein embolization. In the PBD group, the mean age and male:female ratio was 62.94 ± 8.77 years and 25:11, respectively. In the non-PBD group, these values were 65.77 ± 10.05 years and 27:20, respectively. The study flow chart is shown in **Figure 1**, and the patients’ clinical details are shown in **Supplementary Table 1**.

In the PBD group, the initial bilirubin level was 332.83 ± 98.31 μmol/L (31.00–470.92 μmol/L), and the preoperative bilirubin level was 108.68 ± 51.24 μmol/L (7.60–209.90 μmol/L). In the non-PBD group, the initial bilirubin or preoperative bilirubin level was 125.16 ± 110.00 μmol/L (5.20–438.30 μmol/L). Compared with the initial bilirubin level, the preoperative bilirubin level showed a significant decline in the PBD group (*P*<0.001). The initial bilirubin level was significantly higher in the PBD than non-PBD group (*P*<0.001). No significant difference was found in the preoperative bilirubin level between the two groups (*P*=0.368).

### 3.2 Liver volume measurement

**Figure 2:**
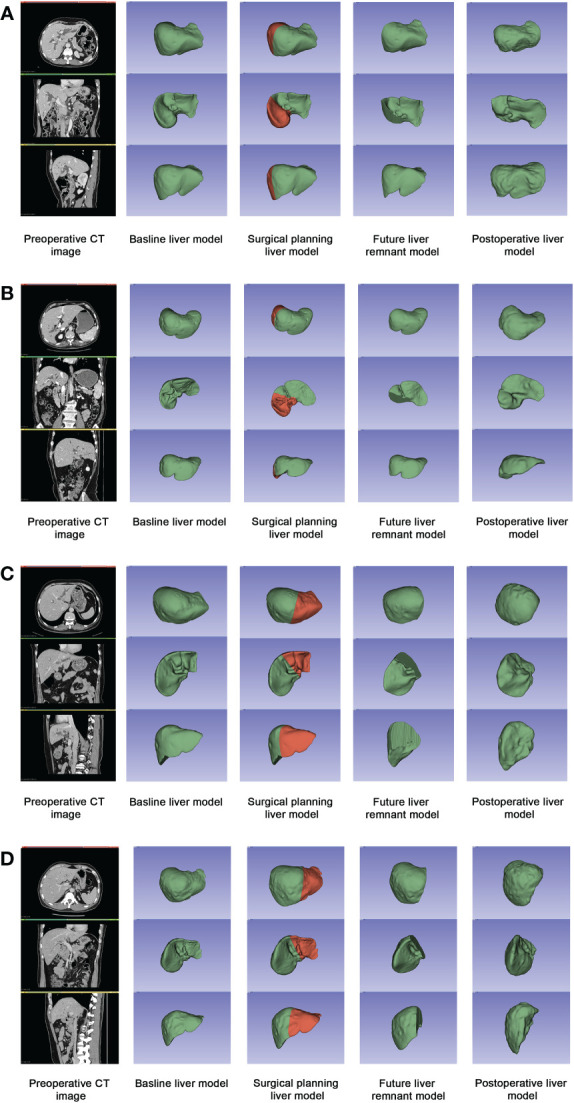
Four sets of three-dimensional images including a preoperative CT image, baseline liver model, surgical planning liver model, FLR model, and postoperative liver model. **(A)** Patient A in PBD group: the initial bilirubin was 467.1μmol/L, and the level of preoperative bilirubin was 199.2μmol/L. The rate of liver regeneration was 28.19 mL/wk, with a regeneration of 27.74% in 9.80wk; **(B)** Patient B in PBD group: the initial bilirubin was 462.9μmol/L, and the level of preoperative bilirubin was 37.5μmol/L. The rate of liver regeneration was 125.61 mL/wk, with a regeneration of 16.30% in 1.57w; **(C)** Patient C in non-PBD group: the preoperative bilirubin was 160.9μmol/L. The rate of liver regeneration was 11.97mL/wk, with a regeneration of 8.78% in 7.43w; **(D)** Patient D in non-PBD group: the preoperative bilirubin was 59.7μmol/L. The rate of liver regeneration was 78.73mL/wk, with a regeneration of 15.80% in 2.29w.

Among all patients, the mean time of liver regeneration (from operation to follow-up CT) was 4.65 ± 4.77 weeks (1–20.86 weeks), the mean V_BLV_ was 1547.17 ± 407.69 mL, the mean future liver remnant volume (V_FLR_) was 983.60 ± 264.91 mL, and the mean V_PRO_ was 1153.43 ± 281.32mL. Compared with the V_FLR_, there was a significant increase in V_PRO_ (*P*<0.001), and the liver regeneration volume accounted for 15.74% ± 9.34% of the SLV. In the PBD group, the liver volume proliferated 186.36 ± 100.18 mL within 5.38 ± 5.66 weeks (*P*<0.001), and liver regeneration accounted for 17.20% ± 8.62% of the SLV. In the non-PBD group, the liver volume increased 157.17 ± 106.48 mL within 4.09 ± 3.92 weeks (*P*<0.001), with 14.62% ± 9.81% regeneration. The changes in liver volume are shown in **Table 1**. Four sets of three-dimensional images including a preoperative CT image, baseline liver model, surgical planning liver model, FLR model, and postoperative liver model are shown in **Figure 2**.

**Table 1:** Changes in liver volume according to PBD in hilar cholangiocarcinoma.

	Baseline liver volume (mL)	Future liver remnant volume (mL)	Duration (wk)	Postoperative liver volume (mL)	Liver volume changes (mL)	Liver volume change, %	Rate of liver regeneration	*P* value
PBD group (n=36)	1676.57 ± 478.71	989.97 ± 299.95	5.38 ± 5.66	1176.33 ± 330.25	186.36 ± 100.18	17.20 ± 8.62	59.77 ± 36.75	*P*<0.001
Non-PBD group (n=47)	1448.06 ± 314.31	978.72 ± 237.88	4.09 ± 3.92	1135.89 ± 239.51	157.17 ± 106.48	14.62 ± 9.81	55.32 ± 33.83	*P*<0.001
All (n=83)	1547.17 ± 407.69	983.60 ± 264.91	4.65 ± 4.77	1153.43 ± 281.32	169.83 ± 104.19	15.74 ± 9.34	57.25 ± 34.98	*P*<0.001

### 3.3 Rate of liver growth

Among all patients, the mean liver regeneration rate was 57.25 ± 34.98 mL/week, with rapid growth of 71.44 ± 31.85 mL/week in the first 4 weeks. The liver volume increase slowed to 42.93 ± 26.06 mL/week from 4 to 8 weeks. After 8 weeks, the rate of liver growth was 17.89 ± 10.37 mL/week. The liver regeneration rate within 4 weeks (71.44 ± 31.85 mL/week, n=54) was significantly higher than that after 4 weeks (30.84 ± 23.49 mL/week, n=29) (*P*<0.001); likewise, growth was significantly higher in the first 8 weeks (65.24 ± 32.72 mL/week, n=69) than after 8 weeks (17.89 ± 10.37 mL/week, n=14) (*P*<0.001).

**Figure 3:**
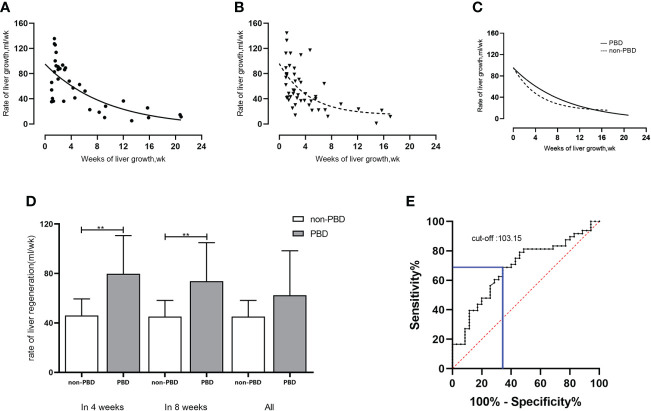
Rate of liver regeneration rate in patients with hilar cholangiocarcinoma undergoing hemi-hepatectomy. **(A-C)** Changes in liver regeneration rate in PBD group and non-PBD group and comparison between the two groups; **(D)** Comparison of liver regeneration rate in patients with initial bilirubin level of >200 μmol/L (within 4 weeks/within 8 weeks/all) between the non-PBD group and the PBD group; **(E)** Cut-off value of preoperative bilirubin level (μmol/L) based on Youden’s index. **P < 0.01.

No significant difference was found in the liver regeneration rate between the PBD group (59.77 ± 36.75 mL/week) and the non-PBD group (55.32 ± 33.83mL/week) (*P*=0.569). Within 4 weeks, the mean rate of liver growth in the PBD group (79.77 ± 30.90 mL/week, n=22) was higher than that in the non-PBD group (65.71 ± 31.69 mL/week, n=32) (*P*=0.112). Within 8 weeks, the mean liver regeneration rate in the PBD group (73.74 ± 31.23 mL/week, n=27) was higher than that in the non-PBD group (59.78 ± 32.86 mL/week, n=42) (*P*=0.112). **Figures 3A-C** shows the changes in the liver regeneration rate with time in the two groups, with each point showing the liver regeneration rate of individuals, as well as a comparison between the two groups.

### 3.4 Univariate and multivariate analyses of factors associated with liver growth rate

The univariate analysis showed that the clinical indicators significantly affecting the liver regeneration rate (*P*<0.05) were body weight (B=1.133, *P*=0.001), body mass index (B=3.241, *P*=0.007), long-term alcohol drinking (B=30.184, *P*=0.010), pathological vascular or neurological invasion (B=18.840, *P*=0.014), preoperative bilirubin level (B=−0.107, *P*=0.013), weeks of liver regeneration (B=−4.294, *P*<0.001), V_PRO_ (B=0.038 *P*=0.005), and V_FLR_ (B=0.032, *P*=0.026).

The factors with a *P* value of <0.05 in the univariate regression analysis were analyzed by multivariate regression analysis. The preoperative bilirubin level (B=−0.191, *P*=0.014), weeks of liver regeneration (B=−0.732, *P*<0.001), V_PRO_ (B=1.151, *P*<0.001), and V_FLR_ (B=−0.969, *P*<0.001) independently affected the liver regeneration rate (**Table 2**).

**Table 2:** Univariate and multivariate analysis of factors of rate of liver growth.

Variable	Univariate analysis *P*-value	Multivariate analysis *P*-value
Weight	0.001	0.504
BMI	0.007	0.303
Drinking history	0.010	0.303
Vascular or nerve invasion	0.014	0.291
Preoperative bilirubin	0.013	0.014
Weeks of liver regeneration	0.001	<0.001
Postoperative liver volume	0.005	<0.001
Future liver remnant volume	0.026	<0.001

### 3.5 Analysis of preoperative factors affecting liver regeneration rate

#### 3.5.1 Effect of PBD on liver regeneration in patients with HC and severe jaundice

For patients with an initial bilirubin level of >200 μmol/L (n=45), the initial bilirubin in the PBD group was significantly higher than that in the non-PBD group (349.05 ± 73.00μmol/L, n=34 vs. 285.15 ± 72.25μmol/L, n=11, respectively; *P*=0.015). The liver regeneration volume and ratio in PBD group (183.51 ± 93.93mL, 17.01 ± 8.24%) was higher than that in non-PBD group (106.23 ± 44.32mL, 10.49 ± 4.45%) by linear regression analysis (*P*=0.012, *P*=0.016). The liver regeneration rate was higher in the PBD group (62.39 ± 36.05 mL/week, n=34) than in the non-PBD group (45.11 ± 13.16 mL/week, n=11) (*P*=0.129). Within 4 weeks, the liver regeneration rate was significantly higher in the PBD group (79.77 ± 30.90 mL/week, n=22) than in the non-PBD group (46.00 ± 13.53 mL/week, n=10) (*P*=0.003). Within 8 weeks, the liver regeneration rate was significantly higher in the PBD group (73.74 ± 31.23 mL/week, n=27) than in the non-PBD group (45.11 ± 13.16 mL/week, n=11) (*P*=0.006). The comparison of the two groups is shown in **Table 3** and **Figure 3D**.

**Table 3:** Comparison of PBD and non-PBD in patients with hilar cholangiocarcinoma whose initial bilirubin > 200μmol/L.

Parameters	Level	PBD (n=34)	Non-PBD (n=11)	P value
Age (yrs)		63.32 ± 8.89	61.45 ± 12.23	0.584
Gender	Male	24 (70.59)	9 (81.82)	0.734
	Female	10 (29.41)	2 (18.18)	
Initial bilirubin (μmol/L)		349.05 ± 73.00	285.15 ± 72.25	0.015
Preoperative bilirubin (μmol/L)		114.06 ± 47.35	285.15 ± 72.25	<0.001
Postoperative liver insufficiency	with	5 (14.71)	6 (54.55)	0.008
	without	29 (85.29)	5 (45.45)	
Liver volume	Baseline liver volume (mL)	1669.67 ± 456.05	1546.72 ± 431.89	0.436
	Future liver remnant volume (mL)	989.77 ± 307.56	1008.60 ± 228.63	0.853
	Duration (wk)	4.84 ± 5.35	2.45 ± 1.10	0.019
	Postoperative liver volume (mL)	1173.28 ± 332.64	1114.83 ± 217.68	0.589
	Liver volume changes (mL)	183.51 ± 93.93	106.23 ± 44.32	0.012
	Liver volume change, %	17.01 ± 8.24	10.49 ± 4.45	0.016
	Rate of liver regeneration	62.39 ± 36.05	45.11 ± 13.16	0.023

#### 3.5.2 Obtaining optimal preoperative bilirubin level based on liver regeneration rate

Taking the mean liver regeneration rate of 57.25 ± 34.98 mL/week in all 83 patients as the boundary value, patients with a liver regeneration rate of ≤57.25 mL/week were defined as the lower liver regeneration rate group (n=48), and those with a rate of >57.25 mL/week were defined as the higher liver regeneration rate group (n=35). The preoperative bilirubin cut-off value calculated by Youden’s index was 103.15 μmol/L (**Figure 3E**), with sensitivity of 0.688 and specificity of 0.657.

For patients with preoperative bilirubin ≤103.15μmol/L (n=38), the rate of liver regeneration (71.77 ± 35.99 mL/week), liver regeneration volume (210.27 ± 116.96mL) and ratio (19.18 ± 10.35%) were higher than those with preoperative bilirubin>103.15μmol/L (n=45,45.00 ± 29.25mL/week, 135.69 ± 78.25mL, 12.84 ± 7.34%) (*P*<0.01, *P*<0.01, *P*=0.02).

In the PBD group (n=36), the liver regeneration rate was significantly higher in patients with a preoperative bilirubin level of ≤103.15 than >103.15 μmol/L (76.14 ± 38.96 mL/week, n=20 vs. 46.68 ± 29.72 mL/week, n=16, respectively; *P*=0.015).

### 3.6 Univariate and multivariate analyses of factors affecting postoperative liver insufficiency

Among all 83 patients, 14 developed postoperative liver insufficiency, including 5 of 36 in the PBD group and 9 of 47 in the non-PBD group. Among patients with a preoperative bilirubin level of >200 μmol/L (n=45), the probability of postoperative hepatic insufficiency was significantly lower in the PBD group (5/34) than in the non-PBD group (6/11) (*P*=0.012, odds ratio=0.144, 95%CI=0.031–0.657). When examining the effect of a preoperative bilirubin level of 103.15 μmol/L (cut-off value) on postoperative hepatic insufficiency, we found that patients with a preoperative bilirubin level of >103.15 μmol/L in the logistic regression analysis (13/45) had a higher probability of postoperative liver insufficiency than those with a lower preoperative bilirubin level (1/38) (*P*=0.011, odds ratio=0.067, 95% CI=0.008–0.537).

The factors affecting postoperative liver insufficiency in the univariate analysis included the initial bilirubin level (*P*=0.026), preoperative albumin–bilirubin score (*P*=0.003), whether the preoperative albumin–bilirubin score reached grade 3 (*P*=0.006), bilirubin cut-off value of 103.15 µmol/L (*P*=0.011), whether the ALB was <30g/L (*P*=0.048), and preoperative bilirubin level (*P*=0.026). The multivariate analysis showed that the preoperative bilirubin level significantly affected postoperative liver insufficiency (*P*=0.016, odds ratio=1.016, β=0.016, 95% CI=1.003–1.029) (**Table 4**).

**Table 4:** Univariate and multivariate analysis of factors of postoperative liver insufficiency.

Variable	Univariate analysis *P*-value	Multivariate analysis *P*-value	β	Exp(B)	EXP(B) 95%CI
Initial bilirubin	0.026	0.315	0.004	1.004	0.997-1.011
ALBI score	0.003	0.277	-2.301	0.100	0.002-6.333
ALBI reached Grade3 (yes/no)	0.006	0.708	0.496	1.642	0.123-21.964
Preoperative bilirubin ≤ 103.15μmol/L	0.011	0.302	1.529	4.613	0.253-84.074
ALB below 30g/L (yes/no)	0.048	0.140	2.074	7.953	0.506-125.086
Preoperative bilirubin	<0.001	0.016	0.016	1.016	1.003-1.029

## 4 Discussion

The standard of PBD for patients with HC has long been controversial (27, 28). Recent studies have considered postoperative liver function, postoperative complications, survival time, and mortality as evaluation indicators to explore the best choice of preoperative treatment for patients with HC (29, 30). However, these indicators are not comprehensive enough to reflect the function and potential growth of the postoperative liver. The function of the residual liver after liver resection is difficult to predict, but the change in liver volume is an important indicator of liver function. The speed at which the liver volume increases may also reflect the potential for liver regeneration (31–33). Calculation of the liver volume based on CT images is widely used in clinical practice to assess the size of liver grafts, prevent postoperative liver failure, and predict postoperative mortality (34–36). Lee et al. showed that the change in liver volume can be used as an effective tool to evaluate the effect of stent implantation in patients with cholangiocarcinoma (10). Because the V_PRO_ of patients with HC changes dynamically with time, we used the rate of liver growth to evaluate the postoperative liver regeneration status and prognosis and to explore the influence of preoperative factors on the change in liver volume, with the goal of providing new ideas for clinical treatment in the future. To our knowledge, this is the first clinical study to focus on the change in liver volume and its influencing factors in patients with HC who have undergone hemi-hepatectomy.

The endpoint of liver regeneration time is still controversial. Lee CH et al. studied the regeneration of the liver from 4 to 20 weeks after biliary stent (10); Zhang Y et al. explored liver volume change in donors and recipients from 0.5 to 6 months after liver transplantation (37); and Gong WF et al. analyzed the liver growth in hepatocellular carcinoma patients at 1, 5, 9, and 13 weeks after liver surgery (38). In this study, we retrospectively analyzed the liver volume changes before and after hepatectomy in 83 patients with HC. The follow-up duration ranged from 1 to 20.86 weeks. Compared with V_FLR_, V_PRO_ increased significantly (*P*<0.001) (increase of 169.83 ± 104.19 mL, which accounted for 15.74% ± 9.34%). A more rapid liver regeneration rate was found within 4 weeks or 8 weeks than after 4 weeks or 8 weeks (*P*<0.001), suggesting that liver regeneration mainly occurs in the early stage after liver resection; this is consistent with the results reported by Lee et al. (10). **Figure 2** shows that the liver morphologically increased in volume after hepatectomy, which may be attributed to the fulfillment of liver function needs, the stimulation by surgery, the relief of biliary obstruction, or other factors. Therefore, this study suggests that the increase in liver volume is a response to the regenerative capacity of the liver. The liver regeneration rate differs among individual patients, which may be explained by the patient’s preoperative state, operative approach, function of the liver, and other factors. We therefore believe that the liver regeneration rate may be used as an effective index to appraise postoperative hepatic function.

The preoperative bilirubin level is an essential factor affecting postoperative liver regeneration (39, 40), and hyperbilirubinemia is considered an independent risk factor for mortality and complications after hepatectomy (29, 41). A recent study showed that obstructive jaundice can induce the proliferation and activation of hepatic stellate cells, resulting in up-regulation of transforming growth factor-β1 mRNA and inhibition of hepatocyte growth factor mRNA in the liver, thus causing delayed liver regeneration after liver resection in rats (42). Kim et al. retrospectively analyzed 112 living donors who underwent right hepatectomy and proposed that liver regeneration can be predicted by combining the preoperative serum total bilirubin level, residual liver volume, and CT texture analysis and that V_FLR_ is an independent predictor of liver regeneration (43). Through univariate and multivariate analyses of factors affecting the liver regeneration rate, we found that a higher preoperative bilirubin level (B=−0.191, *P*=0.014), larger V_FLR_ (B=−0.969, *P*<0.001), longer liver regeneration time (B=−0.732, *P*<0.001), and smaller V_PRO_ (B=1.151, *P*<0.001) led to a lower postoperative liver regeneration rate. These results may suggest that a high preoperative bilirubin level is associated with poor preoperative liver function, which reduces the potential for liver regeneration; that large V_FLR_ indicates the need for smaller liver resection, also reducing the potential for liver regeneration; that the liver regenerative potential decreases over time; and that small V_PRO_ indicates poorer liver regeneration outcomes and predicts poorer liver regeneration potential. Interestingly, our analysis of factors affecting postoperative liver insufficiency showed that preoperative bilirubin was an independent factor affecting postoperative hepatic insufficiency (*P*=0.016, odds ratio=1.016, β=0.016, 95% CI=1.003–1.029). As the preoperative bilirubin level increased, the probability of postoperative hepatic insufficiency also increased. Higher preoperative bilirubin may lead to poor preoperative liver function, which will affect the recovery of postoperative liver function. Therefore, the preoperative bilirubin level may be an important indicator that affects the liver regeneration rate and postoperative liver function.

Whether to perform biliary drainage in the preoperative management of patients with HC is controversial. Many Asian centers advocate routine extensive biliary drainage to reduce jaundice and improve liver function before surgery (21, 44–46). However, it has also been suggested that catheter-implanted metastatic cancer caused by preoperative percutaneous transhepatic biliary drainage is not an uncommon complication (47). Ramanathan et al. proposed that preoperative PBD is associated with increased postoperative complications (48). Our study compared the efficacy of PBD in patients with HD who underwent hemi-hepatectomy from the perspective of the liver regeneration rate. Interestingly, no significant difference in the liver regeneration rate was found between the PBD and non-PBD group (*P*=0.569). We found that although the rate of liver growth was higher in the PBD than non-PBD group within 4 weeks or 8 weeks, the difference was not statistically significant (<4 weeks, *P*=0.112; <8 weeks, *P*=0.084) by further comparing the difference in the liver regeneration rate between the two groups in the early stage of liver regeneration.

Patients with severe jaundice usually require preoperative biliary drainage, but the cut-off value is controversial (41, 49, 50). Cai et al. considered 218.75 μmol/L as the cut-off value of total bilirubin for PBD by retrospectively analyzing 218 patients with HC (49). Another study suggested that biliary drainage is required when the bilirubin level reaches 171.0 μmol/L, considering the patients’ condition and surgical extent (50). Hemming et al. performed palliation of jaundice before liver resection for patients whose preoperative bilirubin level was >85.5 μmol/L (51). Wronka et al. proposed that patients with a total bilirubin level of ≥102.6 μmol/L who underwent PBD may have lower rates of mortality and severe complications (41). We analyzed the liver growth in patients with an initial bilirubin level of >200 μmol/L based on expert consensus in China and the research reported by Laurent (20). The liver regeneration volume and ratio in PBD group were both significantly higher than that in non-PBD group (*P*=0.012, *P*=0.016). During the early postoperative period, the rate of liver growth was significantly higher in the PBD than non-PBD group within 4 or 8 weeks (≤4 weeks, *P*=0.003; ≤8 weeks, *P*=0.006). Notably, among patients with a preoperative bilirubin level of >200 μmol/L, the probability of postoperative liver insufficiency was significantly higher in the non-PBD than PBD group (*P*=0.012). For patients with HC who have severe jaundice (200 μmol/L) and are candidates for hepatectomy, preoperative jaundice palliation may improve liver function and maintain a better liver regeneration potential after the operation, thus enhancing liver regeneration in the early postoperative period. In patients who do not undergo PBD, however, poor liver function caused by severe jaundice may increase the risk of postoperative liver insufficiency.

The recommended optimal preoperative bilirubin level for patients with HC after jaundice palliation widely ranges among recent studies (51–54). Some studies suggest that PBD should be performed to reduce the bilirubin level to 34.2 to 53.1 μmol/L before hepatectomy (1, 45, 53). In a study from Hong Kong, She et al. retrospectively analyzed 90 patients with HC and found that a preoperative bilirubin level of <75 μmol/L can significantly reduce the blood transfusion volume required during the operation and significantly improve patients’ postoperative survival rate (55). Cannon et al. advocated three-segment hepatic drainage to reduce the serum bilirubin level to <171.0 μmol/L to improve remnant liver function (52). The rate of liver regeneration reflects not only changes in liver morphology but also liver function to a certain extent. To our knowledge, no other studies have explored the cut-off value of preoperative bilirubin using the rate of liver regeneration as an outcome. Our study indicated that the preoperative bilirubin cut-off value affecting the liver regeneration rate was 103.15 μmol/L. Patients with preoperative bilirubin ≤103.15 μmol/L exhibited better liver regeneration rate (*P*<0.01), regeneration volume and ratio (*P*<0.01, *P*=0.02), compared to patients with preoperative bilirubin greater than that. Interestingly, we further verified its influence on postoperative hepatic insufficiency and found that the probability of postoperative liver insufficiency in patients with HC was higher in those with a preoperative bilirubin level of >103.15 μmol/L than ≤103.15 μmol/L (*P*=0.011). When we examined the effect of a preoperative bilirubin level of ≤103.15 μmol/L on the liver regeneration rate in the PBD subgroup, we found that the postoperative liver regeneration rate was significantly higher in patients with a bilirubin level of ≤103.15 μmol/L than >103.15 μmol/L (*P*=0.015). These results may help to select the optimal operative time for hepatectomy in patients with HC. Better postoperative liver regeneration and a lower incidence of liver insufficiency may be obtained when the preoperative bilirubin level is ≤103.15 μmol/L. With the goal of prioritizing postoperative safety, earlier surgical treatment can prevent tumor progression and reduce the impact of bile loss on patients’ overall health.

This study had several limitations. First, because it was a retrospective study, it had a certain selection bias, and our sample size was too small to perform a more detailed subgroup comparison. Second, the method by which the liver volume was drawn in this study was semi-automatic, and certain manual delineation errors seem unavoidable. At the same time, the V_FLR_ was obtained by dividing the V_BLV_ along the middle hepatic vein, which deviated from the real postoperative residual liver volume to a certain degree.

## 5 Conclusion

Patients with HC usually develop an increase in liver volume after hepatectomy. The rate of liver regeneration may be an effective index to evaluate postoperative liver function. The preoperative bilirubin level may be an independent risk factor affecting the liver regeneration rate and postoperative liver insufficiency. For patients whose initial bilirubin is >200 μmol/L, PBD may enhance liver regeneration and reduce the incidence of liver insufficiency after surgery. Preoperative bilirubin levels ≤103.15 maybe recommended for leading to a better liver regeneration and lower incidence of postoperative hepatic insufficiency.

## Data availability statement

The original contributions presented in the study are included in the article/**Supplementary Material**. Further inquiries can be directed to the corresponding authors.

## Author contributions

HZ: Data curation, Methodology, Validation, Visualization, Writing – original draft, Writing – review & editing. JZ: Conceptualization, Resources, Supervision, Writing – original draft, Writing – review & editing. BL: Project administration, Supervision, Writing – review & editing. XhL: Conceptualization, Data curation, Writing – review & editing. XnL: Validation, Visualization, Writing – review & editing. CZ: Supervision, Validation, Visualization, Writing – review & editing. TG: Data curation, Formal analysis, Writing – review & editing. ZD: Data curation, Formal analysis, Writing – review & editing.
