# Supplementary material for: Dynamic three-dimensional liver volume assessment of liver regeneration in hilar cholangiocarcinoma patients undergoing hemi-hepatectomy
Source: Front Oncol. 2024 Apr 19;14:1375648. doi: 10.3389/fonc.2024.1375648 (PMC11067054; doi:10.3389/fonc.2024.1375648)
Supplement: Supplementary file 1 [file Table_1.docx]

Supplementary Material

# Supplementary Table 1 Baseline clinical characteristics and liver volume changes of patients with hilar cholangiocarcinoma

| Parameters | Level | PBD group (n=36) | Non-PBD group (n=47) | P value |
| --- | --- | --- | --- | --- |
| Age (yrs) |  | 62.94±8.77 | 65.77±10.05 | 0.185 |
| Gender | Male | 25(69.4) | 27(57.4) | 0.263 |
|  | Female | 11(30.6) | 20(42.6) |  |
| Weight (kg) |  | 63.47±12.36 | 64.33±9.16 | 0.728 |
| BMI (kg/m^2^) |  | 22.32±3.31 | 23.60±2.96 | 0.068 |
| Long-term alcohol drinking | yes | 6(16.7) | 4(8.5) | 0.429 |
|  | no | 30(83.3) | 43(91.5) |  |
| Bismuth–Corlette classification | Ⅲa | 14(38.9) | 6(12.8) |  |
|  | Ⅲb | 15(41.7) | 31(66.0) |  |
|  | IV | 7(19.4) | 10(21.2) |  |
| Liver cirrhosis | With | 10(27.8) | 9(19.1) | 0.354 |
|  | without | 26(72.2) | 38(80.9) |  |
| HBV infection | yes | 3(8.3) | 2(4.3) | 0.758 |
|  | no | 33(91.7) | 45(95.7) |  |
| Initial bilirubin (μmol/L) |  | 332.83±98.31 | 125.16±110.00 | <0.001 |
| Preoperative bilirubin (μmol/L) |  | 108.68±51.24 | 125.16±110.00 | 0.368 |
| Albumin (g/L) |  | 33.84±4.45 | 35.54±4.49 | 0.089 |
| Albumin less than 30 g/L | yes | 6(16.7) | 8(17.0) | 0.966 |
|  | no | 30(83.3) | 39(83.0) |  |
| ALBI score |  | -1.58±0.45 | -1.80±0.65 | 0.070 |
| ALBI reach grade 3 | yes | 12(33.3) | 14(29.8) | 0.730 |
|  | no | 24(66.7) | 33(70.2) |  |
| Ast(U/L) |  | 72.06±54.69 | 117.23±86.32 | 0.005 |
| Alt(U/L) |  | 88.19±79.65 | 152.83±98.59 | 0.001 |
| ALP(U/L) |  | 243.89±115.24 | 479.79±328.55 | <0.001 |
| GGT (U/L) |  | 257.78±144.85 | 868.26±609.44 | <0.001 |
| CA199 (U/L) |  | 373.37±785.01 | 344.8±351.65 | 0.825 |
| CEA (ng/mL) |  | 4.27±3.11 | 4.35±5.49 | 0.937 |
| Hemi-hepatectomy | left | 18(50.0) | 37(78.7) | 0.006 |
|  | right | 18(50.0) | 10(21.3) |  |
| Vascular or nerve invasion | with | 18(50.0) | 27(57.4) | 0.500 |
|  | without | 18(50.0) | 20(42.6) |  |
| Postoperative liver insufficiency | with | 5(13.9) | 9(19.1) | 0.526 |
|  | without | 31(86.1) | 38(80.9) |  |
